# Excessive dynamic airway collapse during general anesthesia: a case report

**DOI:** 10.1186/s40981-020-00380-1

**Published:** 2020-09-28

**Authors:** Shunichi Murakami, Shunsuke Tsuruta, Kazuyoshi Ishida, Atsuo Yamashita, Mishiya Matsumoto

**Affiliations:** 1 Department of Anesthesiology, Shuto General Hospital, Kogaisaku 1000-1, Yanai, Yamaguchi, 742-0032 Japan; 2 Department of Anesthesiology, Japan Community Healthcare Organization Tokuyama Central Hospital, Kodacho 1-1, Shunan, Yamaguchi, 745-8522 Japan; 3 Department of Anesthesiology, Yamaguchi University Graduate School of Medicine, Minami-Kogushi 1-1-1, Ube, Yamaguchi, 755-8505 Japan

**Keywords:** Excessive dynamic airway collapse, Asthma, Bronchoscopy, General anesthesia, Positive end-expiratory pressure

## Abstract

**Background:**

Excessive dynamic airway collapse (EDAC) is an uncommon cause of high airway pressure during mechanical ventilation. However, EDAC is not widely recognized by anesthesiologists, and therefore, it is often misdiagnosed as asthma.

**Case presentation:**

A 70-year-old woman with a history of asthma received anesthesia with sevoflurane for a laparotomic cholecystectomy. Under general anesthesia, she developed wheezing, high inspiratory pressure, and a shark-fin waveform on capnography, which was interpreted as an asthma attack. However, treatment with a bronchodilator was ineffective. Bronchoscopy revealed the collapse of the trachea and main bronchi upon expiration. We reviewed the preoperative computed tomography scan and saw bulging of the posterior membrane into the airway lumen, leading to a diagnosis of EDAC.

**Conclusions:**

Although both EDAC and bronchospasm present as similar symptoms, the treatments are different. Bronchoscopy proved useful for distinguishing between these two entities. Positive end-expiratory pressure should be applied and bronchodilators avoided in EDAC.

## Background

High-inspiratory airway pressure is sometimes encountered during general anesthesia with endotracheal intubation and is usually caused by an asthma attack or chronic obstructive pulmonary disease. However, there are some uncommon causes of this phenomenon, one of which is expiratory central airway collapse. In adults, expiratory central airway collapse occurs because of tracheobronchomalacia (TBM), excessive dynamic airway collapse (EDAC), or both [1]. TBM is characterized by weakness of the tracheobronchial cartilaginous structures and manifests as circumferential flaccidity. In contrast, EDAC results from degeneration of the smooth muscle of the posterior membrane of the tracheobronchial tree and is characterized by excessive bulging of the posterior membranes into the airway lumen without collapse of the cartilage [1].

Here, we report a case in which EDAC was incidentally diagnosed during abdominal surgery under general anesthesia. We obtained written informed consent from the patient and her family for publication of this case report.

## Case presentation

**Fig. 1:**
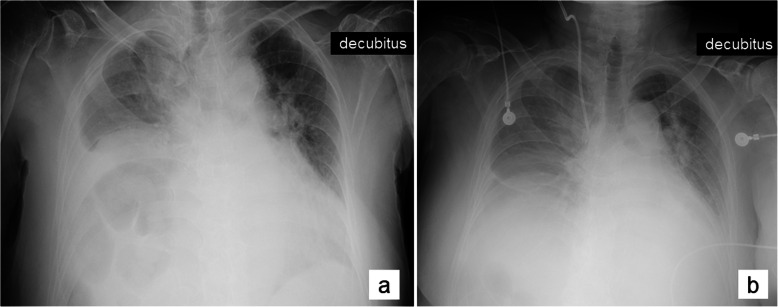
Chest radiographs **a** before surgery and **b** after extubation. **a** Elevation of the right hemidiaphragm, right-sided pleural effusion, and dilated bowel were shown. **b** An increase in the right lung volume was shown

A 70-year-old woman (height 145 cm, weight 65 kg, body mass index 31 kg/m^2^) was transferred to our hospital with fever, abdominal pain, and elevated liver enzymes. Computed tomography (CT) revealed calculous cholecystitis and paralytic ileus. Therefore, an emergent laparotomic cholecystectomy was planned. She had tachypnea but no wheeze or cough. Arterial blood gas analysis showed pH of 7.47, PaO_2_ of 65 mmHg, and PaCO_2_ of 36 mmHg under nasal tube oxygen at 1 L/min. Chest radiography revealed an elevation of the right hemidiaphragm and right-sided pleural effusion (Fig. 1a). Her medical history included schizophrenia, dementia, atrial fibrillation, diabetes mellitus, and untreated asthma. She had no previous history of abdominal or thoracic surgery.

General anesthesia was induced with fentanyl 75 μg, propofol 60 mg, and rocuronium bromide 50 mg under standard monitoring. After endotracheal intubation, anesthesia was maintained with sevoflurane, fentanyl, and rocuronium bromide. Volume-controlled mechanical ventilation was started consisting of 8 mL/kg predicted body weight (respiratory rate 10 breaths per minute with an inspiratory to expiratory ratio of 1:2) without positive end-expiratory pressure (PEEP). Forty-five minutes after induction of anesthesia, we noticed a high peak inspiratory pressure (PIP) of 30 cmH_2_O, bilateral expiratory wheeze, and a shark-fin waveform on capnography without a decrease in percutaneous arterial blood oxygen saturation. There was minimal secretion in the trachea and no kinks in the endotracheal tube. Given the patient’s history of untreated asthma, we assumed she was having an asthma attack and administered 40 μg of procaterol via the endotracheal tube. However, the abnormal waveform and high airway pressure persisted. Although we re-administered procaterol and increased the concentration of inhalational sevoflurane from 1.5 to 2.0%, airway pressure increased further, fluctuating between 27 cmH_2_O and 35 cmH_2_O with each ventilation. Fortunately, she did not develop hypoxia or hypercapnia. Bronchoscopy revealed the collapsed trachea and main bronchi during expiration, indicating expiratory central airway collapse. The addition of PEEP (5 cmH_2_O) and extension of the exhalation time improved her condition promptly. Although the shark fin waveform remained on capnography, the highest PIP decreased from 35 cmH_2_O to 27 cmH_2_O. The surgeons then changed the procedure from cholecystectomy to gallbladder drainage because it was difficult to dissect the gallbladder from the liver bed. Despite lessening of the severity of her condition, peak airway pressure remained high and she was admitted to the postsurgical care unit after surgery without extubation. Operating time was 1 h 26 min and anesthesia time was 2 h 45 min.

**Fig. 2:**
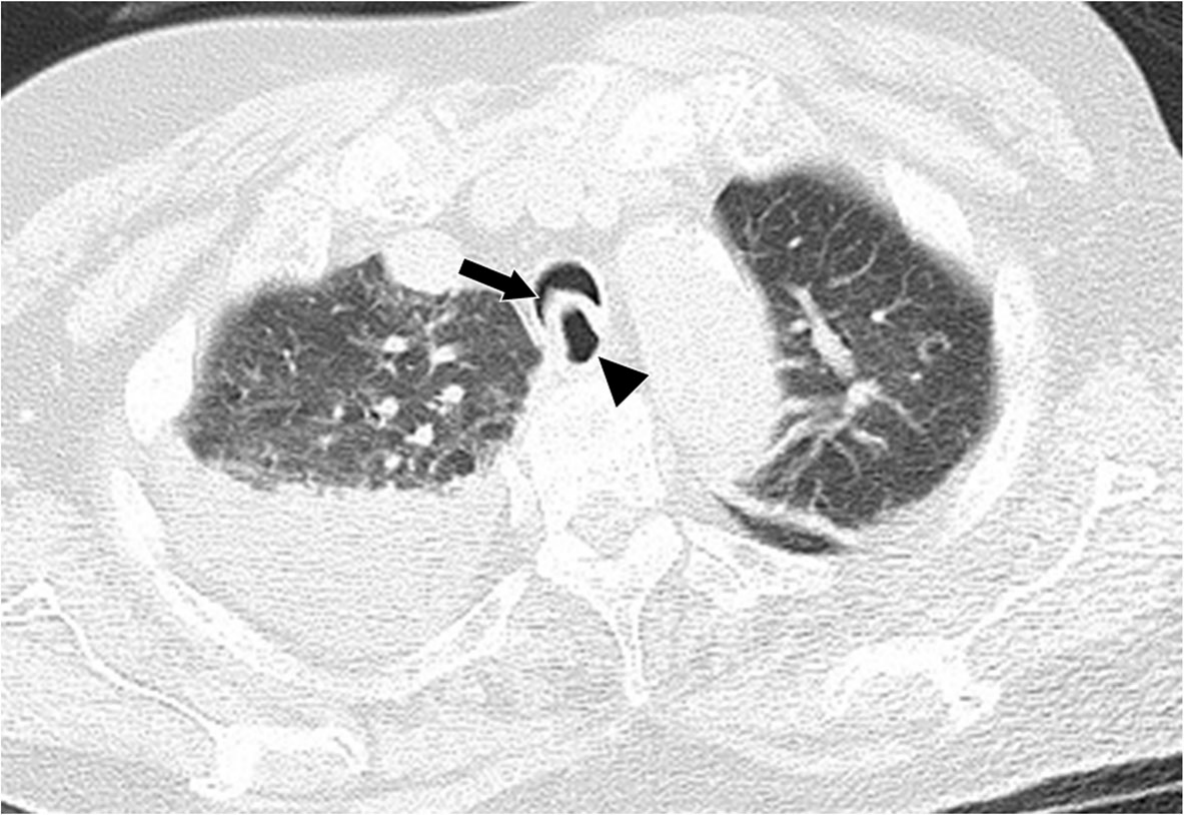
Computed tomography scan of the chest. Computed tomography scan of the chest taken before surgery showing bulging of the posterior membrane (allow) into the airway lumen without collapse of the cartilage at the aortic arch level. An arrowhead indicates the esophagus

After the surgery, we noticed that preoperative non-dynamic CT had captured bulging of the posterior membranes of the trachea and main bronchi into the airway lumen without collapse of the cartilage (Fig. 2). We therefore diagnosed EDAC.

The patient was mechanically ventilated using volume-controlled synchronized intermittent mandatory ventilation with PEEP of 5 cmH_2_O postoperatively. Neither bronchodilators nor corticosteroids were administrated. The airway pressure started to decrease gradually on postoperative day (POD) 5. The patient was extubated after the ventilation mode was changed to continuous positive airway pressure of 5 cmH_2_O on POD 11 (Fig. 1b). The patient did not need further mechanical respiratory support.

## Discussion

EDAC can become unexpectedly severe such that mechanical ventilation becomes difficult and the resulting hypoxia and hypercapnia are life-threatening. Smoking, chronic inflammation, administration of steroids or beta-agonists, and ischemia (caused, for example, by introducing the endotracheal tube, tracheostomy, thyroid tumor, or vascular anomaly) can lead to degeneration of the smooth muscle fibers in the posterior membrane of the tracheobronchial tree [1, 2]. As air travels in the direction of the larynx, the intraluminal pressure decreases from alveolus toward the larynx during expiration. Because the smooth muscle of the trachea and main bronchi is affected with atrophies in EDAC patient, the posterior membrane bulges toward the anterior wall at the part where the intraluminal pressure is below the intrathoracic pressure [1]. One study found the prevalence of EDAC defined by expiratory airway collapse ≥ 75 to be 1.6% (1/62) in non-smokers without obstructive lung disease and 30.7% (62/202) in non-smokers with asthma [3]. Our patient may have been prone to EDAC because of her untreated asthma. The same study also reported the prevalence of EDAC to be 6.8% in a mild asthma group and 69.2% in a severe asthma group [3], indicating that patients with more severe asthma are at higher risk of EDAC.

However, EDAC is not widely recognized by anesthesiologists, probably because it is not clinically serious unless there is a significant narrowing of the airway. The symptoms of airway narrowing are paroxysmal cough, wheeze, and stridor [4]. Those symptoms are also common in asthma and chronic obstructive pulmonary disease. Given the slow progression of EDAC, patients may not notice airway narrowing in everyday life. Although our patient did not have symptoms suggesting airway collapse before surgery during spontaneous breathing, general anesthesia with neuromuscular blockade seemed to cause an increase in intrathoracic pressure, leading to worsening of airway collapse. Furthermore, the choice of sevoflurane and administration of a bronchodilator might have worsened the EDAC by causing relaxation of the smooth muscle.

There has been another report of expiratory central airway collapse after induction of general anesthesia with isoflurane [5]. However, in that case, collapse affected in a limited portion of the trachea, as demonstrated by immediate improvement after the endotracheal tube was advanced 3 cm further down the trachea. In contrast, our case had more severe collapse, extending from the trachea to the main bronchi, as evident from our review of the preoperative CT scans. Our patient was extubated on POD 11. We speculate that improvement in the ileus and a return to spontaneous breathing may have helped reduce the intrathoracic pressure, leading to the improvement of EDAC.

Dynamic bronchoscopy or paired inspiratory-dynamic expiratory CT is often used for the diagnosis of expiratory central airway collapse [4]. In our case, we first detected the airway collapse using bronchoscopy. This incident diagnosis of EDAC was also supported by preoperative non-dynamic CT findings. Non-dynamic CT scans are usually acquired during breath-holding in the end-inspiratory phase, so images of expiratory airway collapse would not usually be obtained. However, the patient’s chest might have been scanned during the expiratory phase due to tachypnea and cognitive decline. Harada et al. [6] also reported incidental detection of EDAC with non-dynamic CT.

PEEP can serve as a useful pneumatic stent if EDAC is suspected during general anesthesia, as in our case. Lyaker et al. [7] reported a case in which EDAC consistently worsened soon after extubation. In severe cases, noninvasive positive pressure ventilation might be necessary after extubation; this was not needed in our patient after extubation, probably because we continued mechanical ventilation until she had recovered satisfactorily. Some reports describe the use of PEEP of 6–10 cmH_2_O as treatment for EDAC not related to anesthesia [6, 8]. In another study, expiratory flow in children with TBM improved at noninvasive positive expiratory pressures of 5, 10, and 15 cmH_2_O but worsened at 20 cmH_2_O [9]. Although these were not cases of intraoperative EDAC, PEEP of 5–15 cmH_2_O may be adequate to improve EDAC that occurs during general anesthesia. In our case, we carefully added PEEP of 5 cmH_2_O and were able to decrease the PIP. However, it is possible that a higher PEEP could have further improved the airway obstruction. If a mechanical ventilator has a function of displaying flow-volume loops, the lowest PEEP value that can get the highest maximum expiratory flow will be a suitable setting. Considering the patient had already presented a hypoxia and a pleural effusion before the operation, we probably should have applied PEEP when mechanical ventilation was initiated.

## Conclusion

EDAC is likely to develop in patients with asthma. Both bronchospasm and worsening EDAC should be considered in a patient with a chronic lung condition such as asthma who develops increased airway pressure, wheezing, and a shark fin waveform on capnography during general anesthesia. Bronchoscopy provides useful for distinguishing between these two entities. As demonstrated in our case, bronchodilators should be avoided in patients with EDAC; instead, it is preferable to increase PEEP and have the patient breathe spontaneously when possible. Finally, it is worth keeping in mind that the preoperative CT scan can occasionally provide useful information about EDAC in patients with a chronic lung condition.

## Data Availability

Not applicable.

## Abbreviations

EDAC: Excessive dynamic airway collapse
TBM: Tracheobronchomalacia
CT: Computed tomography
PEEP: Positive end-expiratory pressure
PIP: Peak inspiratory pressure
POD: Postoperative day
